# Radiographic and Clinical Outcomes of Dual Mobility Total Hip Arthroplasty: A Retrospective Comparative Study from a Tertiary Centre

**DOI:** 10.3390/diagnostics16081241

**Published:** 2026-04-21

**Authors:** Monica Georgiana Roman, Alexandru Lisias Dimitriu, Elisa Georgiana Popescu, Eduard Catalin Georgescu, Liliana Mirea, Razvan Ene, Dragos Ene

**Affiliations:** 1 Department 14 Orthopaedics—Intensive Care, “Carol Davila” University of Medicine and Pharmacy, 050474 Bucharest, Romania; monica-georgiana.roman@drd.umfcd.ro (M.G.R.); elisa-georgiana.popescu@drd.umfcd.ro (E.G.P.); eduard-catalin.georgescu@drd.umfcd.ro (E.C.G.); liliana.mirea@umfcd.ro (L.M.); razvan.ene@umfcd.ro (R.E.); 2 Department of Orthopaedics, Clinical Emergency Hospital, 010024 Bucharest, Romania; 3 Department of Anesthesiology and Intensive Care, Clinical Emergency Hospital, 010024 Bucharest, Romania; 4 Department 10 General Surgery, “Carol Davila” University of Medicine and Pharmacy, 050474 Bucharest, Romania; dragos.ene@umfcd.ro; 5 Department of Surgery, Clinical Emergency Hospital, 010024 Bucharest, Romania

**Keywords:** dual mobility, total hip arthroplasty, radiographic evaluation, implant positioning, acetabular cup orientation, hip instability, revision hip arthroplasty

## Abstract

**Background:** Dual mobility (DM) total hip arthroplasty (THA) was introduced to reduce postoperative instability, one of the most frequent causes of revision after hip replacement. Its use has progressively expanded beyond revision surgery to selected high-risk primary cases; however, comparative data integrating both clinical and radiographic outcomes from real-world tertiary centers remain limited. **Methods:** A retrospective comparative study was conducted including 78 patients who underwent THA with a DM acetabular component between January 2019 and December 2024, and 78 matched controls who received conventional fixed-bearing THA during the same period. Matching criteria were age, sex, and procedure type (primary versus revision). Clinical outcomes were assessed using the Harris Hip Score (HHS) and visual analogue scale (VAS) for pain. Radiographic evaluation focused on component positioning, radiolucent lines, and signs of loosening. Complications and revision rates were compared between groups. **Results:** The mean age was 71 ± 9 years, and 62% of patients were female. Mean follow-up was 38 months. HHS improved from 54 ± 10 preoperatively to 89 ± 8 postoperatively in the DM group (*p* < 0.001), with similar final functional outcomes in the conventional THA group (90 ± 9, *p* = 0.48), and comparable improvement between groups (*p* = 0.62). Radiographic parameters demonstrated stable fixation and appropriate component positioning in both groups, with no significant intergroup differences. The dislocation rate was numerically lower in the DM group (1.3% vs. 5.1%), although this difference did not reach statistical significance (*p* = 0.37). No cases of intraprosthetic dislocation occurred. Overall implant survival free from revision at five years was 96.5% for DM and 94.7% for conventional THA (*p* = 0.47). **Conclusions:** DM THA achieved excellent clinical and radiographic outcomes, with a numerically lower dislocation rate than conventional THA. Mid-term implant survivorship was comparable between groups, supporting DM as a reliable option for improving stability in appropriately selected patients.

## 1. Introduction

THA is widely regarded as one of the most successful procedures in orthopaedic surgery, consistently providing substantial pain relief and functional improvement for patients with end-stage hip disease [1]. Despite these favorable outcomes, postoperative instability remains one of the most challenging complications, with reported dislocation rates ranging from 0.5% to 10% in the general population and increasing to as much as 28% in high-risk or revision settings [1,2,3]. Dislocation has a significant negative impact on patient satisfaction and quality of life and is associated with an increased risk of early revision surgery, higher healthcare costs, and greater overall morbidity [4].

In an effort to reduce instability after THA, the DM was introduced in France by Gilles Bousquet and André Rambert in the late 1970s [5]. This design combines the low-friction principle described by Charnley with an additional mobile polyethylene liner articulating within the acetabular shell [5]. By increasing the effective jump distance and delaying impingement, DM constructs enhance joint stability while preserving an adequate range of motion and maintaining acceptable wear characteristics [6].

Over the past two decades, substantial advances in implant design, metallurgy, and polyethylene processing have further improved the performance of DM cups, significantly reducing the incidence of intraprosthetic dislocation and improving long-term outcomes [7,8].

Initially, DM implants were primarily reserved for revision arthroplasty or for patients at high risk of instability, such as those with neuromuscular disorders, cognitive impairment, or post-traumatic deformities [2]. More recently, their indications have expanded to selected primary THA cases, particularly in elderly and frail patients with reduced soft-tissue tension or altered spinopelvic mechanics [9,10]. Several clinical studies have reported dislocation rates as low as 0–1% and excellent implant survivorship at mid- to long-term follow-up, supporting the growing adoption of this concept in contemporary practice [11,12]. Nevertheless, evidence derived from real-world tertiary orthopaedic and trauma centers—where elective, traumatic, and revision cases frequently coexist—remains limited.

At our institution, DM acetabular components have been increasingly used since 2019 in complex primary and revision THA, with the aim of improving postoperative stability without compromising functional recovery.

In addition to implant design, surgical approach has been shown to influence postoperative stability and functional outcomes in THA. Systematic reviews have demonstrated that different surgical approaches are associated with variable risks of dislocation and complication profiles in both primary and revision settings, highlighting the multifactorial nature of hip stability [13].

The present study therefore aimed to evaluate the indications and clinical outcomes of DM THA in comparison with conventional fixed-bearing THA over a six-year period. Secondary objectives included the assessment of complication rates, dislocation incidence, and implant survivorship in both cohorts, thereby contributing to the ongoing discussion regarding the optimal role of DM constructs in modern hip arthroplasty practice.

## 2. Materials and Methods

### 2.1. Study Design and Setting

This retrospective comparative cohort study was conducted at the Department of Orthopaedics and Traumatology, Clinical Emergency Hospital of Bucharest, a tertiary academic center affiliated with the “Carol Davila” University of Medicine and Pharmacy. The study included all consecutive patients who underwent THA using a DM acetabular component between January 2019 and December 2024. Ethical approval was obtained from the institutional review board prior to data collection.

### 2.2. Study Population

The study included two cohorts: patients who underwent THA using a DM acetabular component and a matched control group treated with conventional fixed-bearing THA during the same study period.

Matching was performed using a nearest-neighbor approach without replacement, based on age (±5 years), sex, and procedure type (primary versus revision THA). When multiple potential matches were available, the closest age match was selected. Although matching was performed in a 1:1 ratio, it was not designed as strict pair-matching for paired statistical analysis; therefore, the two groups were analyzed as independent cohorts.

Baseline variables, including ASA score, Charlson comorbidity index, surgical indication, fixation method, and femoral head size, were systematically recorded and included in the analysis. The distribution of surgical indications was not used as a formal matching criterion but was subsequently compared between groups and found to be similar.

Clinical risk factors for instability, such as neuromuscular disease or spinopelvic imbalance, were not included as matching variables due to incomplete data availability.

### 2.3. Inclusion and Exclusion Criteria

Patients aged 18 years or older who underwent primary or revision THA using either a DM or a conventional fixed-bearing acetabular component were eligible for inclusion in the study. Only patients with complete preoperative and postoperative clinical outcome scores and available radiographic documentation were included. A minimum follow-up duration of 12 months was required for eligibility.

Patients were excluded if they presented with an active periprosthetic joint infection or systemic sepsis at the time of surgery. Additional exclusion criteria included the presence of neoplastic disease involving the pelvis or proximal femur, as well as incomplete clinical or radiographic follow-up or loss to follow-up before 12 months.

### 2.4. Surgical Technique

All procedures were performed or directly supervised by fellowship-trained hip surgeons. A posterior Kocher–Langenbeck approach was used in the majority of cases, with patients positioned in the lateral decubitus position. The choice of fixation (cemented or uncemented) for both the acetabular component and femoral stem was guided by bone quality, patient age, and intraoperative findings.

In the DM cohort, most acetabular components consisted of uncemented hemispherical monoblock cups with highly cross-linked polyethylene mobile liners (Avantage^®^ Dual Mobility Cup System, Zimmer Biomet, Warsaw, IN, USA). In selected revision cases with compromised bone stock, cemented DM components were used.

In a subset of complex revision cases with significant acetabular bone defects, trabecular metal augments or shells were used to restore bone stock, followed by cementation of a DM cup within the reconstructed acetabulum. This technique was selected based on intraoperative assessment of bone loss and implant stability.

All patients received standard perioperative antibiotic prophylaxis, pharmacological thromboprophylaxis according to institutional protocols, and early postoperative mobilization with weight-bearing as tolerated.

### 2.5. Data Collection and Variables

Clinical, surgical, and radiographic data were retrospectively collected from the institutional database and patient records by the study investigators. Clinical variables included patient demographics (age, sex, and body mass index), as well as comorbidity-related parameters assessed using the American Society of Anesthesiologists (ASA) grade [14] and the Charlson comorbidity index [15]. Surgical indications included primary osteoarthritis, femoral neck fracture, post-traumatic arthritis, avascular necrosis, developmental dysplasia, and revision surgery for instability or aseptic loosening.

Functional outcomes were assessed using the Harris Hip Score (HHS) for functional evaluation [16], applied using a Romanian-language version routinely used in clinical practice at our institution, which has not undergone formal validation, and the visual analogue scale (VAS) for pain [17], recorded preoperatively and at final follow-up.

Surgical variables included procedure type (primary versus revision THA), operated side, surgical approach, fixation method (cemented versus uncemented), implant brand, femoral head size, operative time, and length of hospital stay.

Radiographic evaluation was performed using standardized anteroposterior pelvic radiographs obtained at follow-up. Acetabular component inclination and anteversion were assessed using established radiographic methods based on the orientation of the elliptical projection of the acetabular cup, as described by Lewinnek et al. [18] and Murray et al. [19]. Care was taken to ensure appropriate patient positioning, and radiographs with significant pelvic tilt or rotation were excluded when measurements were considered unreliable. To reduce measurement bias, all radiographic assessments were independently performed by two investigators who were not involved in the surgical procedures and were blinded to clinical outcomes. Interobserver agreement for radiographic measurements was high, with an intraclass correlation coefficient (ICC) of 0.91 for acetabular inclination and 0.88 for anteversion. In cases of discrepancy, a consensus reading was obtained. Stem alignment was evaluated on anteroposterior views and categorized as neutral, varus, or valgus based on the relationship between the femoral stem axis and the femoral shaft. Radiographic variables included acetabular cup inclination and anteversion, femoral stem alignment, and the presence of radiolucent lines, osteolysis, or loosening.

Recorded complications included postoperative dislocation, intraprosthetic dislocation, periprosthetic fracture, infection, aseptic loosening, and the need for re-revision. Implant survival was defined as the absence of revision for any reason at final follow-up.

### 2.6. Outcome Measures

The primary outcome measure was the postoperative dislocation rate. Secondary outcomes included changes in HHS and VAS scores, overall complication rate, implant survival at five years, and the distribution of cemented versus uncemented fixation between groups. Implant survival was defined as the absence of revision surgery involving removal or exchange of any prosthetic component (acetabular, femoral, or modular components). Procedures such as irrigation and debridement without component exchange, closed reduction for dislocation, or internal fixation for periprosthetic fracture with retention of the implant were not considered revision events. Revision events were defined a priori and recorded prospectively in the institutional database.

### 2.7. Statistical Analysis

Statistical analyses were performed using IBM SPSS Statistics, version 26.0 (IBM Corp., Armonk, NY, USA). Continuous variables were expressed as mean ± standard deviation (SD) and compared using Student’s *t*-test or the Mann–Whitney *U* test, depending on data distribution. Categorical variables were analyzed using the chi-square test or Fisher’s exact test, as appropriate. Paired *t*-tests were applied for within-group preoperative and postoperative comparisons. Implant survivorship was assessed using Kaplan–Meier analysis, with revision for any reason as the endpoint, and differences between groups were evaluated using the log-rank test. Statistical significance was set at *p* < 0.05.

Artificial intelligence tools (ChatGPT, OpenAI, version GPT-5.3) were used solely for language editing and improvement of clarity and structure. No AI tools were used for data generation, statistical analysis, or interpretation of the results.

## 3. Results

### 3.1. Patient Characteristics

**Figure 1:**
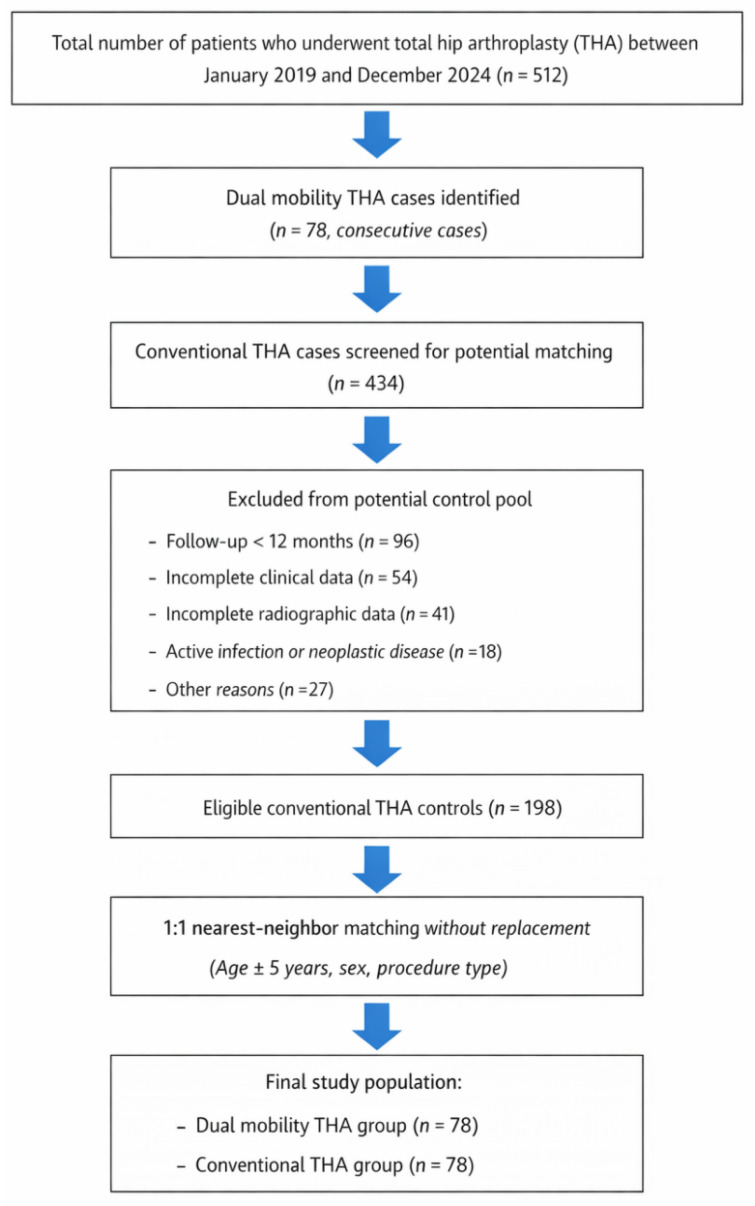
Study flow diagram of patient selection and cohort matching.

A total of 156 patients were included in the final analysis, comprising 78 patients in the DM group and 78 patients in the conventional to THA group. The mean age at the time of surgery was 71 ± 9 years (range, 48–89 years), and 62% of the study population were female. In the DM cohort, 42 procedures were performed as primary THA and 36 as revision THA, with a comparable distribution observed in the conventional THA group (45 primary and 33 revision procedures). The patient selection process is summarized in Figure 1.

The mean body mass index was 28.1 ± 4.3 kg/m^2^, and the mean duration of follow-up was 38 ± 15 months (range, 12–72 months). The indications for THA are summarized in Table 1. In the DM group, the most common indications were primary osteoarthritis (37%), post-traumatic arthritis or femoral neck fracture (23%), revision for recurrent instability (26%), and developmental dysplasia or avascular necrosis (14%).

No statistically significant differences were observed between the two groups with respect to age, sex distribution, body mass index, or surgical indication (all *p* > 0.05).

### 3.2. Surgical Details

All procedures were performed or directly supervised by the same senior hip surgeon, in order to minimize variability related to surgical technique and perioperative decision-making. The posterior Kocher–Langenbeck approach was used in the majority of cases, accounting for 87% of procedures overall, with a similar distribution between the DM and standard THA groups (85% and 88%, respectively). Acetabular fixation was predominantly uncemented in both cohorts, being used in 94% of cases in the DM group and 90% in the standard THA group. Femoral stems were cementless in 83% of patients in the DM group and 78% in the control group, with no statistically significant difference between groups (*p* = 0.42).

The mean operative time was 92 ± 21 min for DM THA and 88 ± 18 min for standard THA (*p* = 0.27). The mean length of hospital stay was comparable between groups, averaging 6.1 ± 2.0 days in the DM group and 5.9 ± 1.8 days in the standard THA group (*p* = 0.58).

In a limited number of revision cases, acetabular reconstruction involved the use of trabecular metal components, followed by cementation of a DM cup. These cases were characterized by substantial bone loss and were managed using advanced reconstructive techniques.

### 3.3. Clinical Outcomes

Both cohorts demonstrated significant postoperative functional improvement. In the DM group, the mean HHS increased from 54 ± 10 preoperatively to 89 ± 8 at final follow-up (*p* < 0.001). Similarly, patients in the standard THA group showed an improvement in HHS from 56 ± 11 preoperatively to 90 ± 9 at last follow-up (*p* < 0.001). No statistically significant difference was observed between groups with respect to the final HHS values (*p* = 0.48).

Pain relief followed a comparable pattern in both cohorts. In the DM group, the mean VAS score decreased from 6.8 ± 1.1 preoperatively to 1.2 ± 0.9 at final follow-up (*p* < 0.001), while in the standard THA group VAS scores improved from 6.5 ± 1.3 to 1.1 ± 0.8 (*p* < 0.001). Overall, both groups achieved significant reductions in pain and substantial functional recovery, with no statistically significant differences between them. Detailed clinical outcomes are presented in Table 2.

### 3.4. Complications and Dislocation

Overall complication rates were low and comparable between the two cohorts, occurring in 11.5% of patients in the DM group and 14.1% in the conventional THA group (*p* = 0.61). Postoperative dislocation occurred in 1 patient (1.3%) in the DM group and in 4 patients (5.1%) in the conventional THA group. Although the dislocation rate was numerically lower in the DM group, this difference did not reach statistical significance (*p* = 0.37). The relative risk of dislocation in the DM group was 0.25 (95% CI: 0.03–2.19), although the wide confidence interval reflects the limited number of events.

The single dislocation observed in the DM group occurred following a revision procedure in an elderly patient with documented spinopelvic imbalance and was successfully managed with closed reduction, without recurrence. No cases of intraprosthetic dislocation were recorded in either cohort.

Early deep infection was documented in three patients (1.9%), including one patient in the DM group and two patients in the conventional THA group. All cases were managed with irrigation and debridement combined with targeted antibiotic therapy, without the need for implant removal. No recurrent or late infections were observed during the follow-up period.

Periprosthetic fractures occurred in two patients (2.6%) in the conventional THA group and in one patient (1.3%) in the DM group. All fractures involved the femoral stem and followed low-energy falls; each case was successfully managed with internal fixation while retaining the acetabular component. No cases of aseptic loosening or liner dissociation were identified in the DM cohort.

### 3.5. Radiographic Findings

Radiographic evaluation demonstrated stable component fixation in the vast majority of cases. Non-progressive radiolucent lines measuring less than 2 mm were observed in 5 patients (6.4%) in the DM group and in 6 patients (7.7%) in the standard THA group. These findings were limited in extent, non-progressive over time, and were not associated with clinical symptoms or implant loosening. No cases of component migration, osteolysis, or aseptic loosening were identified in either group during the follow-up period. Only two patients overall demonstrated minor radiographic changes considered potentially relevant; however, these did not result in clinical deterioration or require further intervention.

Mean acetabular cup inclination was 43.5° ± 5.2° in the DM group and 44.1° ± 5.0° in the standard THA group, while mean cup anteversion measured 20.8° ± 4.3° and 21.1° ± 4.5°, respectively. No statistically significant differences were observed between groups for either parameter (both *p* > 0.05).

Stem alignment was comparable between groups, with the majority of stems positioned in neutral alignment and no significant differences observed between cohorts.

**Figure 2:**
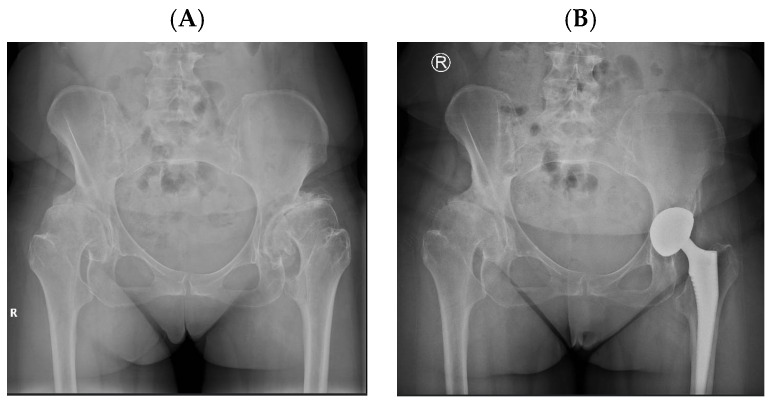
(**A**) Preoperative anteroposterior pelvic radiograph showing advanced hip osteoarthritis secondary to developmental dysplasia. (**B**) Postoperative radiograph following THA with a DM acetabular component.

**Figure 3:**
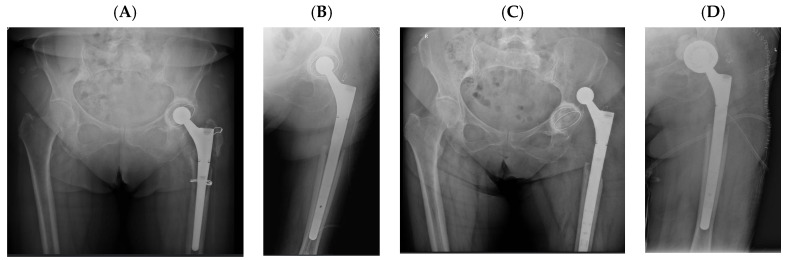
Complex revision case in an adult patient following oncologic resection. (**A**) Initial radiograph; (**B**) postoperative image after femoral reconstruction; (**C**) prosthesis dislocation; (**D**) revision with DM component.

**Figure 4:**
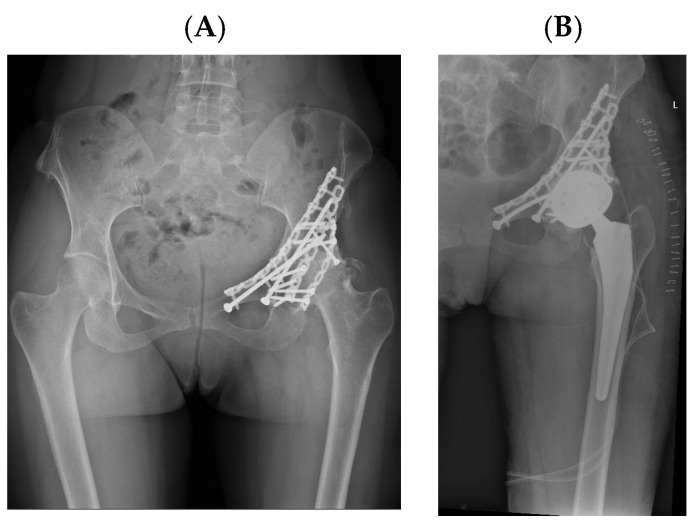
Post-traumatic osteoarthritis in an adult female patient following previous acetabular fracture fixation. (**A**) Preoperative radiograph; (**B**) postoperative radiograph after THA with a DM component.

**Figure 5:**
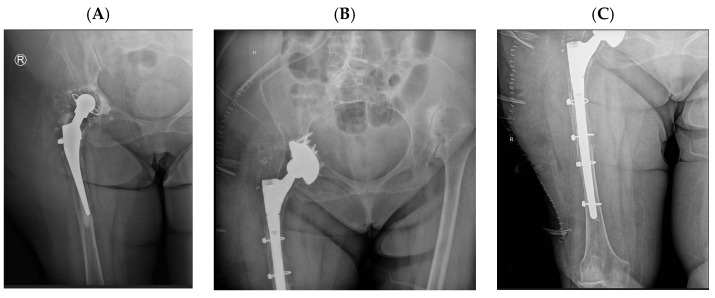
(**A**) Radiograph showing a right THA with a cemented acetabular component and femoral stem, complicated by femoral stem loosening and partial acetabular cup loosening. (**B**,**C**) Postoperative radiographs following revision surgery.

Representative cases illustrating the application of DM constructs in different complex clinical scenarios are presented in Figure 2, Figure 3, Figure 4 and Figure 5. Figure 3, Figure 4 and Figure 5 represent illustrative cases outside the analytic cohort and are included for educational purposes.

### 3.6. Implant Survival

At a mean follow-up of 38 months, implant survival free from revision was 96.5% in the DM group and 94.7% in the standard THA group, with no statistically significant difference between cohorts (*p* = 0.47, log-rank test).

Revision was defined as any surgical procedure involving removal or exchange of a prosthetic component. During the study period, only a small number of revision procedures were recorded, primarily due to recurrent instability and aseptic loosening.

Importantly, cases of infection managed with irrigation and debridement without component removal, as well as periprosthetic fractures treated with internal fixation while retaining the implant, were not classified as revision events.

**Figure 6:**
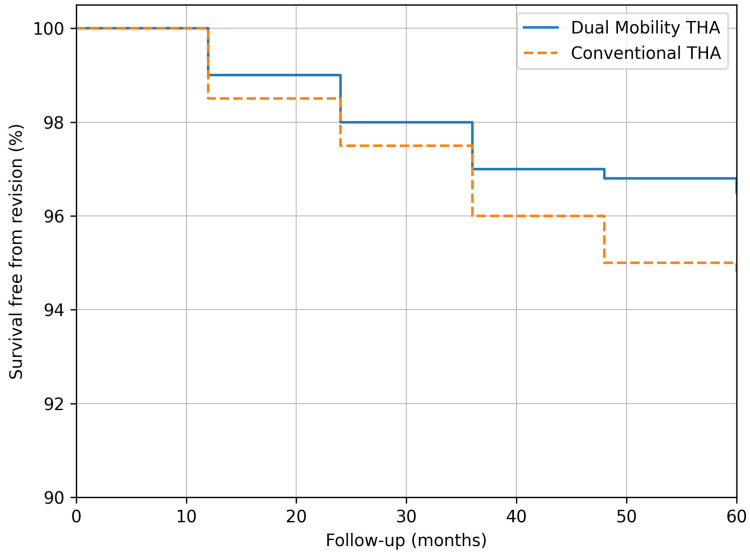
Kaplan–Meier survival analysis comparing DM and conventional THA over a five-year follow-up.

The Kaplan–Meier survival analysis (Figure 6) demonstrates comparable mid-term revision-free survivorship between the DM and conventional THA cohorts. Kaplan–Meier survival curves were constructed with 95% confidence intervals. Due to the limited number of events, formal comparison between groups should be interpreted with caution.

Revision events are detailed in Table 3.

The Kaplan–Meier curves demonstrate implant survival free from revision for any cause in both groups. At 60 months, survival was 96.5% for the DM group and 94.7% for the conventional THA group.

The difference between curves was not statistically significant (*p* = 0.47, log-rank test). Both cohorts showed excellent short- to mid-term survivorship, with slightly higher stability and fewer revisions in the DM group.

## 4. Discussion

The present study evaluated the indications and clinical outcomes of DM THA in comparison with conventional fixed-bearing THA in a tertiary orthopaedic and trauma center over a six-year period. Our findings demonstrate that DM implants provide excellent functional outcomes and were associated with a numerically lower rate of postoperative dislocation in a heterogeneous population including both primary and revision cases. Importantly, these outcomes were achieved without compromising mid-term implant survivorship, which was comparable between the two groups.

It should be noted, however, that the selection of DM components was influenced by perceived instability risk based on clinical and intraoperative judgment. This introduces potential confounding by indication, which should be considered when interpreting differences in instability outcomes between groups.

### 4.1. Stability and Dislocation Rates

Postoperative instability remains one of the most frequent causes of early failure after THA, accounting for up to 20% of all revision procedures in some national registries [11]. The DM concept was developed to address this issue by increasing the effective jump distance and delaying impingement, thereby reducing the risk of dislocation without compromising range of motion.

In the present cohort, the postoperative dislocation rate was 1.3% in the DM group compared with 5.1% in the standard THA group. Although this difference did not reach statistical significance, DM constructs were associated with a lower numerical rate of dislocation. This finding should be interpreted with caution, particularly given the limited number of events and the retrospective design. While these results suggest a potential protective effect of DM implants in terms of instability, they do not provide definitive evidence of superiority. The inclusion of both primary and revision procedures reflects real-world practice but introduces heterogeneity, as revision cases are inherently associated with higher complication and instability risks. This aspect should be considered when interpreting the observed differences in dislocation rates between groups.

These findings are consistent with previous reports demonstrating lower instability rates with DM constructs [20,21,22,23]. Philippot et al. [24] reported a 0.9% dislocation rate at ten years in primary DM THA, and Levin et al. [25] found similarly favorable outcomes in revision cases, highlighting the potential value of DM particularly in elderly, frail, or neurologically impaired patients [26,27]. DM components were frequently selected in cases perceived to be at higher risk of instability based on intraoperative and clinical judgment; however, the proportion of primary and revision procedures was comparable between groups, reflecting the real-world heterogeneity of a tertiary referral population.

### 4.2. Functional and Radiographic Outcomes

Both treatment groups achieved substantial improvements in functional outcome and pain relief at final follow-up, confirming that the DM mechanism does not compromise functional restoration. The mean postoperative HHS observed in the DM cohort (89 points) is consistent with values reported for contemporary DM implants in previous studies by Batailler et al. [28] and De Martino et al. [29].

Radiographically, all components remained stable, with only minor non-progressive radiolucency, again in line with contemporary literature [30].

### 4.3. Implant Survival and Mechanical Behavior

In the present series, the five-year implant survival rate was 96.5% in the DM group and 94.7% in the conventional THA group, with no statistically significant difference between cohorts. These findings indicate that DM constructs provide mid-term implant durability comparable to that of conventional acetabular designs, while offering a lower observed rate of postoperative instability in this high-risk population. Large registry-based studies from France and Scandinavia have reported similar results, demonstrating comparable long-term survivorship of DM and standard THA, with revision most commonly related to infection or aseptic loosening rather than implant design failure [30,31]. Notably, no cases of intraprosthetic dislocation were observed in our cohort. This finding is consistent with contemporary reports indicating that the incidence of this historically relevant complication has been reduced to below 1% with modern DM systems [21].

These findings are supported by modern implant design improvements, including highly cross-linked polyethylene and optimized articulation geometry, which contribute to reduced wear and improved mechanical performance [32].

This concept is further illustrated by an illustrative case from our institutional experience (Figure 4), which is not part of the analyzed cohort, in which femoral stem loosening and significant acetabular bone loss required combined reconstructive strategies. The use of a trabecular metal acetabular component with a cemented DM cup allowed restoration of hip biomechanics while simultaneously addressing instability risk, highlighting the versatility of DM constructs in revision-like environments.

In addition to implant-related factors, patient-related characteristics remain important determinants of postoperative outcomes. Our results are in line with recent multicenter data showing that comorbidities and overall systemic health status significantly influence complication rates after THA, including the risk of infection, early reoperation, and functional recovery [33].

Concerns related to metal-on-metal articulations, including metal ion release and adverse local tissue reactions, have historically emphasized the importance of implant material selection and biocompatibility in THA. In this context, contemporary bearing designs, including DM constructs, have evolved toward improved safety profiles and reduced complication rates [34].

### 4.4. Cemented Versus Uncemented Fixation

Both cemented and uncemented fixation strategies were used, with no significant differences observed in outcomes, supporting the versatility of DM constructs across fixation techniques. This finding supports existing evidence indicating that the DM concept is compatible with both cemented and cementless constructs, provided that appropriate component positioning is achieved [35]. In revision settings or in patients with compromised bone quality, cemented DM cups have been shown to represent a reliable reconstructive option [36].

In the most complex revision scenarios encountered in our cohort, this concept was extended to include reconstruction with trabecular metal components combined with cemented DM cups. This strategy allowed restoration of acetabular integrity while simultaneously addressing instability risk. Although applied in a limited number of cases, it highlights the versatility of DM constructs in managing severe bone loss in tertiary referral settings.

### 4.5. Clinical Implications

Our experience indicates that DM acetabular components are particularly valuable in several well-defined clinical scenarios. These include elderly and frail patients with reduced soft-tissue tension, revision THA performed for instability or aseptic loosening, and post-traumatic or dysplastic hips in which accurate cup positioning and restoration of offset are technically challenging.

In addition, DM constructs appear especially beneficial in patients with spinopelvic imbalance, a condition known to increase the risk of impingement and recurrent dislocation. Incorporating these considerations into preoperative planning may substantially reduce the risk of postoperative instability without compromising functional outcomes or implant longevity.

Overall, our findings support the use of DM constructs as a reliable option in both primary and revision THA, particularly in patients at increased risk of instability; however, these results should be interpreted with caution given the study design and limited statistical power.

### 4.6. Limitations

This study has several limitations that should be acknowledged. First, its retrospective design is inherently associated with a risk of selection bias. Although a matching strategy was applied to reduce confounding, the study was not designed as a strictly paired analysis, and residual imbalance between groups cannot be entirely excluded.

Second, the duration of follow-up, with a mean of 38 months, provides relevant mid-term data but does not allow assessment of long-term implant survival, polyethylene wear, or late mechanical complications.

Third, radiographic evaluation was based on standard plain radiographs rather than advanced imaging modalities such as computed tomography. Radiographic assessment was therefore limited, without advanced imaging or formal interobserver reliability analysis, which may have introduced measurement variability. In addition, functional outcomes were primarily assessed using the HHS, without inclusion of additional patient-reported outcome measures.

Another important limitation relates to the observational design and the potential for residual confounding by indication. DM components were more frequently used in complex or high-risk cases, including revision procedures and patients with compromised soft-tissue balance. These included patients with clinical risk factors for instability, such as spinopelvic imbalance or neuromuscular conditions, which were not formally accounted for in the matching process.

Moreover, the inclusion of both primary and revision THA reflects real-world practice in a tertiary referral setting but introduces clinical heterogeneity, as revision cases are inherently associated with higher risks of complications and instability. The relatively limited sample size precluded adequately powered subgroup analyses, and therefore potential differences between primary and revision procedures should be interpreted with caution.

Although matching was performed for age, sex, and procedure type, and additional baseline variables such as ASA score, Charlson comorbidity index, surgical indication, fixation method, and femoral head size were recorded and compared between groups, no formal multivariable adjustment or propensity-based analysis was performed. Therefore, the results should be interpreted in the context of this inherent selection bias.

Furthermore, the study was underpowered to detect statistically significant differences in relatively rare outcomes such as dislocation, and therefore the results should be interpreted with caution.

Despite these limitations, the present study represents one of the larger single-center series comparing DM and conventional THA in a heterogeneous, real-world tertiary orthopaedic setting.

## 5. Conclusions

DM THA was associated with excellent functional outcomes and a numerically lower rate of postoperative dislocation compared with conventional THA. However, this difference did not reach statistical significance and should be interpreted with caution, particularly in the context of the retrospective design and limited number of events.

Mid-term implant survivorship was comparable between groups, without an apparent increase in mechanical complications or infection rates. These findings suggest that DM acetabular components represent a reliable option for improving hip stability in both primary and revision arthroplasty, particularly in appropriately selected patients at increased risk of instability. Further studies with larger sample sizes are needed to confirm these observations.

## Figures and Tables

**Table 1:** Patient demographics and indications for surgery.

Variable	DM Group (*n* = 78)	THA Group (*n* = 78)	Statistical Test	*p*-Value
Age (years, mean ± SD)	71 ± 9	70 ± 8	*t*(154) = 0.61	0.54
Female sex, *n* (%)	48 (61.5%)	50 (64.1%)	χ^2^(1) = 0.10	0.75
BMI (kg/m^2^, mean ± SD)	28.1 ± 4.3	27.8 ± 4.6	*t*(154) = 0.41	0.68
ASA score (mean ± SD)	2.4 ± 0.6	2.3 ± 0.7	*t*(154) = 0.92	0.36
Charlson comorbidity index (mean ± SD)	4.8 ± 1.9	4.5 ± 1.7	*t*(154) = 1.02	0.31
Procedure type				
Primary THA, *n* (%)	42 (53.8%)	45 (57.7%)	χ^2^(1) = 0.22	0.64
Revision THA, *n* (%)	36 (46.2%)	33 (42.3%)	χ^2^(1) = 0.22	0.64
Indication for surgery				
Osteoarthritis	29 (37.2%)	31 (39.7%)	χ^2^(1) = 0.22	0.64
Post-traumatic/fracture	18 (23.1%)	16 (20.5%)	χ^2^(1) = 0.22	0.64
Instability (revision)	20 (25.6%)	17 (21.8%)	χ^2^(3) = 1.35	0.72
Dysplasia/AVN	11 (14.1%)	14 (17.9%)	χ^2^(3) = 1.35	0.72
Fixation method				
Cemented	28 (35.9%)	30 (38.5%)	χ^2^(2) = 0.48	0.79
Uncemented	42 (53.8%)	40 (51.3%)	χ^2^(2) = 0.48	0.79
Hybrid	8 (10.3%)	8 (10.3%)	χ^2^(2) = 0.48	0.79
Femoral head size				
28 mm	6 (7.7%)	22 (28.2%)	χ^2^(2) = 0.48	0.79
32 mm	10 (12.8%)	28 (35.9%)	χ^2^(2) = 0.48	0.79
36 mm	14 (17.9%)	20 (25.6%)	χ^2^(3) = 12.1	0.72
≥40 mm (dual mobility articulation)	48 (61.5%)	8 (10.3%)	χ^2^(3) = 12.1	0.007

Values are presented as mean ± standard deviation or number (percentage). Abbreviations: DM = dual mobility. THA = total hip arthroplasty. SD = standard deviation. BMI = body mass index. ASA = American Society of Anesthesiologists. AVN = avascular necrosis.

**Table 2:** Clinical outcomes.

Variable	DM Group (*n* = 78)	THA Group (*n* = 78)	Statistical Test	*p*-Value
HHS preoperative (mean ± SD)	54 ± 10	56 ± 11	*t*(154) = 1.02	0.31
HHS final follow-up (mean ± SD)	89 ± 8	90 ± 9	*t*(154) = 0.71	0.48
HHS improvement (mean ± SD)	35 ± 9	34 ± 10	*t*(154) = 0.49	0.62
VAS preoperative (mean ± SD)	6.8 ± 1.1	6.5 ± 1.3	*t*(154) = 1.24	0.22
VAS final follow-up (mean ± SD)	1.2 ± 0.9	1.1 ± 0.8	*t*(154) = 0.58	0.57

Pre- and postoperative comparisons within each group were performed using paired *t*-tests. Intergroup comparisons were performed using independent *t*-tests or Mann–Whitney *U* tests, as appropriate. HHS—Harris Hip Score [16]; VAS—Visual Analogue Scale [17].

**Table 3:** Causes of revision in the DM and conventional THA groups.

Cause of Revision	DM Group (*n* = 78)	THA Group (*n* = 78)
Recurrent instability	1	2
Aseptic loosening	1	2
Infection (component removal)	0	0
Periprosthetic fracture (revision)	0	0
Total revisions	2 (2.6%)	4 (5.1%)

## Data Availability

The data supporting the findings of this study are available from the corresponding author upon reasonable request. The data are not publicly available due to ethical and privacy restrictions.

## Abbreviations

The following abbreviations are used in this manuscript:

DM	Dual Mobility
THA	Total Hip Arthroplasty
HHS	Harris Hip Score
VAS	Visual Analogue Scale
ASA	American Society of Anesthesiologists
BMI	Body Mass Index
AVN	Avascular Necrosis
